# Adaptive algorithm utilizing acceptance rate for eliminating noisy epochs in block-design functional near-infrared spectroscopy data: application to study in attention deficit/hyperactivity disorder children

**DOI:** 10.1117/1.NPh.5.4.045001

**Published:** 2018-10-11

**Authors:** Stephanie Sutoko, Yukifumi Monden, Tsukasa Funane, Tatsuya Tokuda, Takusige Katura, Hiroki Sato, Masako Nagashima, Masashi Kiguchi, Atsushi Maki, Takanori Yamagata, Ippeita Dan

**Affiliations:** a Hitachi Ltd., Research and Development Group, Center for Exploratory Research, Saitama, Japan; b Jichi Medical University, Department of Pediatrics, Shimotsuke, Japan; c International University of Health and Welfare, Department of Pediatrics, Shiobara, Japan; d Chuo University, Research and Development Initiatives, Applied Cognitive Neuroscience Laboratory, Tokyo, Japan; e Jichi Medical University, Center for Development of Advanced Medical Technology, Shimotsuke, Japan

**Keywords:** functional near-infrared spectroscopy, motion and physiological noises, adaptive algorithm, acceptance rate, personalized evaluation, controlled rejection, attention deficit/hyperactivity disorder

## Abstract

Functional near-infrared spectroscopy (fNIRS) signals are prone to problems caused by motion artifacts and physiological noises. These noises unfortunately reduce the fNIRS sensitivity in detecting the evoked brain activation while increasing the risk of statistical error. In fNIRS measurements, the repetitive resting-stimulus cycle (so-called block-design analysis) is commonly adapted to increase the sample number. However, these blocks are often affected by noises. Therefore, we developed an adaptive algorithm to identify, reject, and select the noise-free and/or least noisy blocks in accordance with the preset acceptance rate. The main features of this algorithm are personalized evaluation for individual data and controlled rejection to maintain the sample number. Three typical noise criteria (sudden amplitude change, shifted baseline, and minimum intertrial correlation) were adopted. Depending on the quality of the dataset used, the algorithm may require some or all noise criteria with distinct parameters. Aiming for real applications in a pediatric study, we applied this algorithm to fNIRS datasets obtained from attention deficit/hyperactivity disorder (ADHD) children as had been studied previously. These datasets were divided for training and validation purposes. A validation process was done to examine the feasibility of the algorithm regardless of the types of datasets, including those obtained under sample population (ADHD or typical developing children), intervention (nonmedication and drug/placebo administration), and measurement (task paradigm) conditions. The algorithm was optimized so as to enhance reproducibility of previous inferences. The optimum algorithm design involved all criteria ordered sequentially (0.047 mM mm of amplitude change, 0.029  mM  mm/s of baseline slope, and 0.6×interquartile range of outlier threshold for each criterion, respectively) and presented complete reproducibility in both training and validation datasets. Compared to the visual-based rejection as done in the previous studies, the algorithm achieved 71.8% rejection accuracy. This suggests that the algorithm has robustness and potential to substitute for visual artifact-detection.

## 1 Introduction

Functional near-infrared spectroscopy (fNIRS) noninvasively measures the product of concentration changes of cerebral hemoglobin ($\text{oxygenated}/O_{2}\mathrm{Hb}$ and deoxygenated/HHb) and optical length using two or more near-infrared spectra (650 to 950 nm).^1^^–^^4^ It measures blood-related signals that indirectly correspond to brain activation (i.e., neurovascular coupling theorem).^5^^,^^6^ After >20 years of development,^7^ fNIRS has gained much attention in broad applications including studies of neuropsychiatry and cognition in infants and children.^8^^–^^10^ Compared to other functional imaging techniques, it shows significant advantages in terms of system flexibility (without confinement or head restrainers) and motion tolerability since probes are tightly adhered on the scalp.^11^^–^^13^ However, pediatric studies have shown that infants and children sometimes become restless, thus causing probe detachment and unavoidable occurrence of motion artifacts. As the name implies, this is especially true for studies of children with attention deficit/hyperactivity disorder (ADHD).

The issue of detecting and removing motion artifacts has been extensively studied; however, there is no golden approach to detect and remove them. Although direct and complete data rejection might be infeasible due to limited and insufficient sample number,^14^ motion correction techniques with supplementary measurements or corrective algorithms also pose technical limitations in measurement and insufficient practicability in analysis.^15^^–^^18^ Implementing an additional system or devices to detect motions (e.g., an accelerometer) on infants or children can complicate the measurement system and induce inconvenience for subjects, which can result in higher probability that motion artifacts will occur. Moreover, correction algorithms^14^^,^^19^^–^^21^ such as filtering (e.g., wavelet, Kalman), regression–interpolation [e.g., spline,^22^ adapted hemodynamic response function (HRF) model], and component reduction require estimated parameters that seemingly lead to noise over-fitting and severe modification of signal waveform.

In the conventional method, motion artifacts are recognized by researchers through the visual observation of sudden and discontinuous signal changes. This recognition may be adjusted while trying to maintain the sufficient sample number; hence we introduced a concept to adaptively identify and reject motion-affected signals in this paper. This concept emphasized the automatic trade-off potential between personal noise level and rejection control rate. By initially setting the acceptable rejection rate, one or more pre-established noise criteria (see Sec. 2) were serially arranged as an artifact evaluator. Particular criteria causing excessive rejections were neglected and the artifact detection–rejection was controlled by the remaining criteria. We performed a simulation using a synthetic noisy signal to confirm our concept. We then used studied fNIRS datasets^23^^,^^24^ measured in typically developing (TD) children and those with ADHD in order to assess the algorithm feasibility. The former corresponds to the general situation of fNIRS measurements of children. On the other hand, the latter represents technically challenging but executable situation as fNIRS has turned out to be one of the preferable techniques to evaluate ADHD patients with a restless characteristic.^25^^–^^28^ Both training and validation steps tried to optimize the reproducibility of group analysis result and rejection accuracy. The dynamic relationship among criteria type-parameters was explored in this adaptive algorithm.

## 2 Materials and Methods

### 2.1 Datasets

**Fig. 1:**
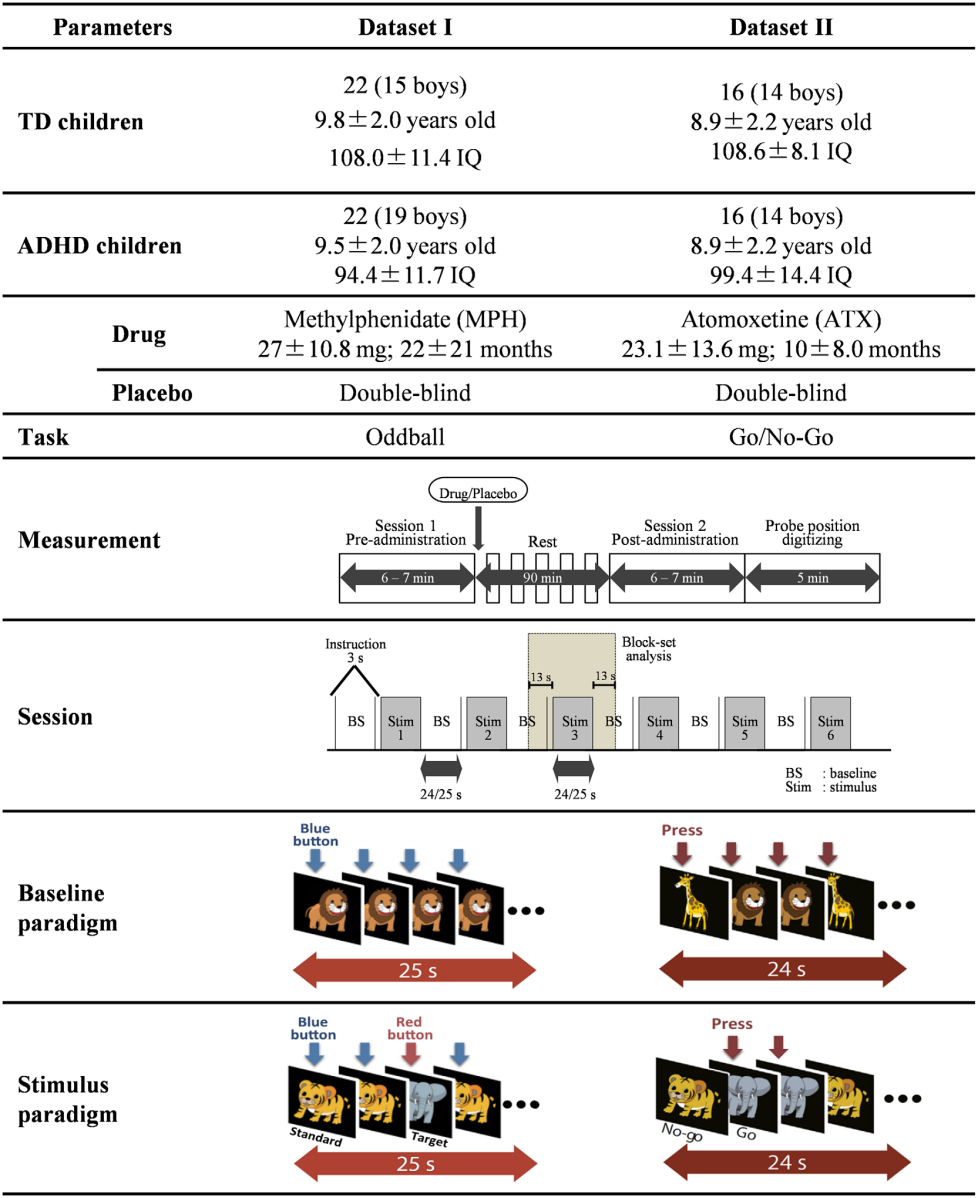
Subject and measurement information.

We used previous datasets collected for studies of neuropharmacological effects in children with ADHD. Details regarding the experimental protocol are described elsewhere;^23^^,^^24^ however, we summarize related information as follows. Right-handed subjects were classified into two datasets as presented in detail in Fig. 1.^23^^,^^24^ All ADHD children were clinically diagnosed using the DSM-IV criteria and age-matched TD children were referred to as controls. All ADHD children were nonmedicated naive and had been treated using either methylphenidate (MPH) or atomoxetine (ATX) medication. In the Wechsler Intelligence Scale of Children—Third Edition (WISC-III), the full IQ scores of subjects were all over 70. The pharmacological effects were assessed in randomized, double-blind, placebo-controlled, and crossover studies. Medication and placebo administrations were only performed in ADHD children, not in TD children. Therefore, TD children underwent only a measurement session while ADHD children completed four sessions—before/after medication and before/after placebo intakes.

Measurements were performed using the multichannel fNIRS system ETG-4000 (Hitachi Medical Corporation, Japan) with two wavelengths of near-infrared spectra (695 and 830 nm). Eight emitters and seven detectors were alternately positioned on a probe holder (3×5 arrangement) and two probe holders (one for each hemisphere) were put on the head covering both the hemispheres of lateral prefrontal and inferior parietal cortices in accordance with previous studies.^29^^–^^33^ The measurement site (also known as the channel) was located in the middle of the emitter and the detector, resulting in 22 channels for each probe holder. Probe position was digitized for all subjects after the first measurement session, and the channel coordinates were spatially registered to MNI space.^34^^–^^39^ A measurement session consisted of six repetitive series of baseline, instruction, and stimulus periods lasted for about 5 min in total. The stimulus involved two tasks called visual-based oddball (OB) and go/no-go (GNG) to examine subject competences in sustained attention and inhibitory function, respectively.^10^^,^^23^^,^^24^^,^^40^^,^^41^ A subject was assigned to perform either an OB or GNG task. Both tasks were created and subject responses were collected by E-Prime 2.0 (Psychology Software Tools).

These studies were approved by the Ethics Committees of Jichi Medical University Hospital and the International University of Health and Welfare. All subjects provided written parent consent. These studies were designed in accordance with the latest version of the declaration of Helsinki. The collaboration between Jichi Medical University Hospital and Hitachi, Ltd. was reviewed by an internal board at Central Research Laboratory, Hitachi, Ltd.

### 2.2 Preprocessing and Activation Analysis

Signal preprocessing was done on the MATLAB-based software Platform for Optical Topography Analysis Tools (POTATo, Hitachi Ltd., Research and Development).^42^ To begin with, the detected optical density data were converted into the product of hemoglobin concentration change and optical path length,^43^^,^^44^ which we define as $\Delta C_{O2\mathrm{Hb}}$, $\Delta C_{\mathrm{HHb}}$, and $\Delta C_{\mathrm{Hb}\text{-}\text{total}}$ in mM mm because of the variation of path length over brain regions.^45^ This calculation is based on the modified Beer–Lambert law as described elsewhere.^43^^,^^46^ Obtained continuous ΔC signals were preprocessed with a first-degree polynomial fitting and band-pass filter using cut-off frequencies of 0.01 and 0.8 Hz to remove baseline drift and heartbeat pulsation, respectively. By adapting the block design, continuous signals were compartmentalized into six epochs similar to the number of repetitive series of baseline, instruction, and stimulus. An epoch included 13 s of prestimulus (10-s baseline and 3-s instruction), 24 to 25 s of stimulus, and 13 s of poststimulus periods. These epochs served as the input for the rejection algorithm. After applying the rejection algorithm, the baseline amplitudes of the remaining epochs were normalized to zero before performing the activation analysis. Activation was defined in a channel-wise manner as the average of the remaining epochs during the activated period (i.e., 4 s after stimulus onset to the end of the stimulus).

### 2.3 Design of Rejection Algorithm

The algorithm was constructed in accordance with the following two processes: (1) noise identification and (2) rejection judgment.

**Fig. 2:**
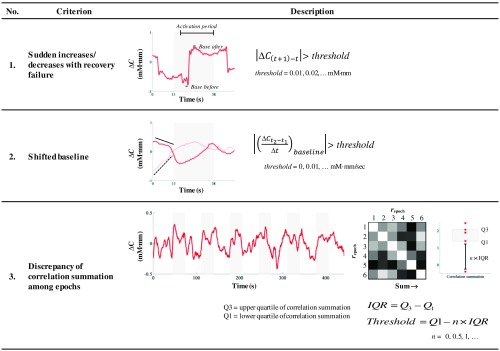
Noise criteria.

*Noise identification*. We adopted three typical noise criteria (Fig. 2). Criterion 1 controlled sudden increases/decreases in signal amplitude with recovery failure to the initial amplitude level. The time needed for recovery became longer as the changes in amplitude became greater. To identify this kind of noise, the algorithm calculated the amplitude change between two sampling points (0.1 s) and marked the onset and ending time when signal amplitude rose above a threshold level. The algorithm then recognized the base levels of the signal amplitude before and after the over-threshold change (e.g., 1-s interval). If the amplitude base level was shifted over 0.2 mM mm, recovery failure was detected and acknowledged as noise (and vice versa). Although noise detected by criterion 1 is likely caused by motion artifacts, criterion 2 managed physiological noises mainly due to spontaneous low-frequency oscillations. These oscillations produce spurious signals^47^^–^^49^ during the baseline and stimulus intervals. Because assessing stimulus interval likely introduced assumptions such as HRF; therefore, the algorithm only examined the baseline slopes (e.g., 10-s prestimulus and 3-s instruction intervals, 13 s in total) using linear fitting estimation. If the baseline slopes are greater than the threshold, those epochs will be labeled as noisy epochs. We believe that noisy epochs have low similarity with other epochs. Hence, criterion 3 evaluated correlation among epochs. For example, in order to understand the relationship between epoch 1 and each of the other epochs, the algorithm summed the correlation between epoch 1 and each of the other epochs. If epoch 1 had noises, the correlation summation was low. Epochs having outlier correlation sums were defined as noisy epochs. Outlier range was determined depending on a threshold defined by an adaptive constant×interquartile range (IQR). In a holistic manner, both criteria 1 and 2 examine noise level according to the global threshold across a subject, whereas criterion 3 specifically controlled the personal threshold.

**Fig. 3:**
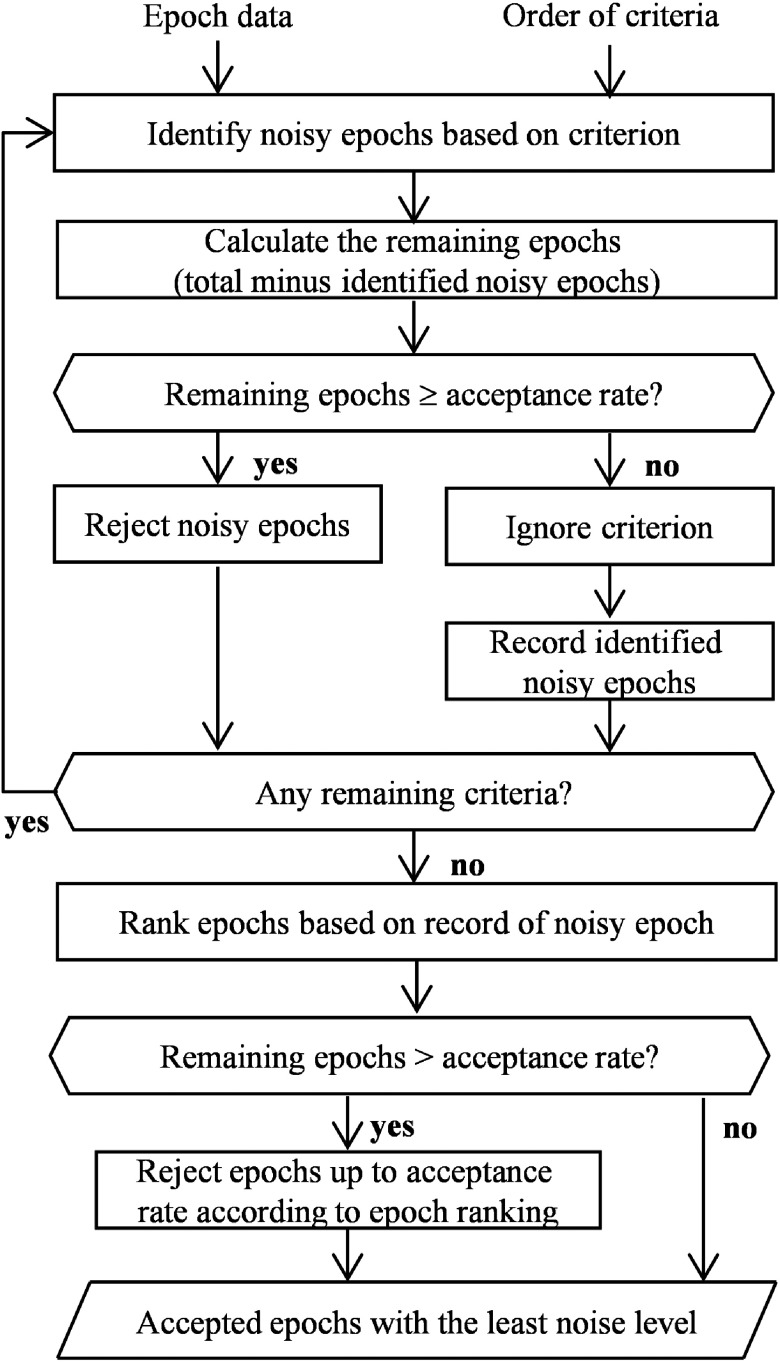
Algorithm process design.

*Rejection judgment*. As mentioned above, the main concepts of the current algorithm were personalized evaluation and controlled rejection. In conventional noise rejection methods, the judgment driven by only the fixed threshold would lead to excessive rejection. We would like to avoid the data loss while maintaining the objectivity of noise identification and rejection. Therefore, the algorithm proposed that the rejection judgment was based on both dataset and individual noise levels. The algorithm process design is shown in Fig. 3 in detail. (1) The algorithm identifies that the presence of noisy epochs according to the above criteria. (2) The algorithm calculates how many epochs remain after rejection. (3) The algorithm inspects whether the number of remaining epochs was greater than or equal to the acceptance rate, which corresponds to the minimum expected value of remaining epochs. (4) The algorithm executes the rejection if process 3 is met. Otherwise, the algorithm terminates the rejection if the above condition (i.e., process 3) is unmet; however, it still records detected noisy epochs. (5) The algorithm applies the next noise criterion only on the remaining/nonrejected epochs. Processes 1 to 5 are repetitively performed until all set criteria have been examined. Epochs are ranked depending on the occurrence of nonrejected noisy epochs (hereafter noise levels). Furthermore, this ranking is rechecked. If the number of remaining epochs is greater than the acceptance rate and some remaining epochs have been identified as noisy epochs, the rejection proceeds in accordance with the epoch noise level ranking. The rejection continues until the number of remaining epochs is equal to the acceptance rate. This algorithm design tries to introduce a fuzzy approach, in which the epochs are labeled not only as noisy or noise-free but also as less noisy epochs. Simply put, the algorithm attempts to reduce noise rejection to a tolerable level while maintaining the least noisy epoch and sufficient epoch number.

### 2.4 Dataset Characteristic and Quality

**Fig. 4:**
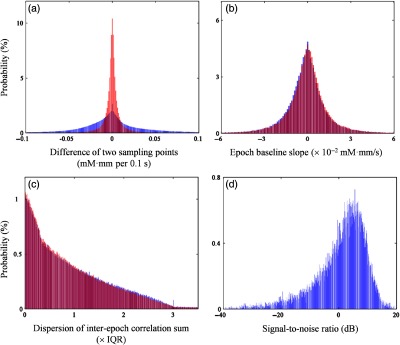
Dataset characteristics: difference of (a) two sampling points, (b) epoch baseline slope, (c) dispersion of interepoch correlation sum, and (d) quality.

For better performance, the algorithm required the optimization of parameters including the criteria thresholds and the acceptance rate. Before doing so, we evaluated the occurrence probability of noise in accordance with the above criteria in the real datasets to determine the parameter ranges. Figure 4(a)–4(c) shows the real dataset characteristics in all channels and signal types (i.e., $\Delta C_{O2\mathrm{Hb}}$ and $\Delta C_{\mathrm{HHb}}$). There was no remarkable effect from the preprocessing in criteria 2 and 3 [Figs. 4(b) and 4(c); blue and red histograms for without and with preprocessing, respectively]. However, preprocessing affected the distribution of two sampling-point differences with greater kurtosis and smaller standard deviation. According to the characteristic observation, the optimization limits for criteria 1 to 3 were selected as 95% and 97.5% of the one- and two-tail accumulative distributions, respectively. Therefore, the optimization ranges for criteria 1 and 2 were 0.01 to 0.05 mM mm with 0.001 in steps, and 0 to 3×IQR with 0.1 in steps for criterion 3. Furthermore, the distribution of signal-to-noise ratio [SNR; Eq. (1) in dB] was evaluated to represent the quality of datasets. Figure 4(d) shows the distribution of SNR in raw datasets with negative skewness calculated as follows:

$$\mathrm{SNR}=20\times \log_{10}\frac{\mu_{\Delta C_{(t)\to t\;\text{activation}}}}{\sigma_{\Delta C_{(t)\to t\;\text{baseline}}}},$$ (1)

where $t_{\text{activation}}$ is the activation interval from 4 s after stimulus onset to stimulus end and $t_{\text{baseline}}$ is the period from 10 s before stimulus to stimulus onset. The datasets have been analyzed in prior studies and the information of visual rejection was known. On the basis of the visual rejection result, around 35% of samples used all epochs (i.e., six) without any rejection, >60% of data underwent either one- or two-epoch rejections, and <1% of data had three remaining epochs.

### 2.5 Simulation of Random Epoch Rejection

**Fig. 5:**
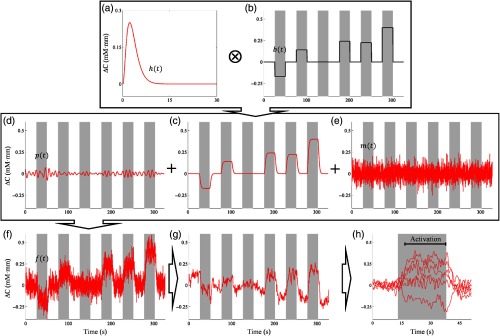
Generation of artificial signal without synthetic noise models. (a) HRF is convolved with the (b) boxcar function resulting in (c) task-related hemodynamic response signal. The fNIRS synthetic signal is composed of the (c) task-related signal, (d) physiological, and (e) machine noises. After (g) preprocessing, (h) the epoch interval from continuous signal is extracted. Gray areas represent the stimulus interval.

To confirm our method, we performed a simulation analysis with random epoch rejection. First, the artificial signals were generated in the following two ways:

1. Synthetic noise-free model (Fig. 5). An HRF [h(t), Fig. 5(a)] was created on the basis of the gamma function^50^ [Eq. (2)] with parameters τ and n as 1.08 and 3, respectively
  $$h(t)={\left(\frac{t}{\tau }\right)}^{n-1}\frac{e^{\left(-\frac{t}{\tau }\right)}}{(n-1)!\tau }.$$ (2)

The HRF was then convolved with the boxcar function [i.e., b(t), Fig. 5(b)] adapting the block-design stimulus as our real datasets (i.e., six trials, 25 to 26 s of pre- and poststimulus, and 24 to 25 s of stimulus intervals). The boxcar amplitude was varied (μ=0.14 to 0.3; σ=0 to 0.16; and $\frac{\mu }{\sigma }=0.85$, 1, 2) to reflect the variances of stimulus response. The mean of the boxcar amplitude was selected in the 0.14 to 0.30 range reflecting the characteristics of real datasets. The variation of $\frac{\mu }{\sigma }$ was determined following the average (i.e., 0.85) and maximum (i.e., 2) values of $\frac{\mu }{\sigma }$ in the real TD data with positive activations. A physiological task-independent component was added as random Gaussian noises with 0 mean and 1/6 standard deviation with fourth-order Butterworth filtering, in the range of 0.08 to 0.15 Hz [i.e., p(t), Fig. 5(d)].^51^ To represent machine noises, random Gaussian noises with 0 mean were also added [i.e., m(t), Fig. 5(e)]. The machine noise variance was adjusted to simulate the SNR parameter. SNR parameter was varied from −40 to 20 dB with an interval of 2 dB. Therefore, the artificial signals [i.e., f(t), Fig. 5(f)] were the combination of normalized h(t)*b(t) [Fig. 5(c)], p(t), and m(t).

**Fig. 6:**
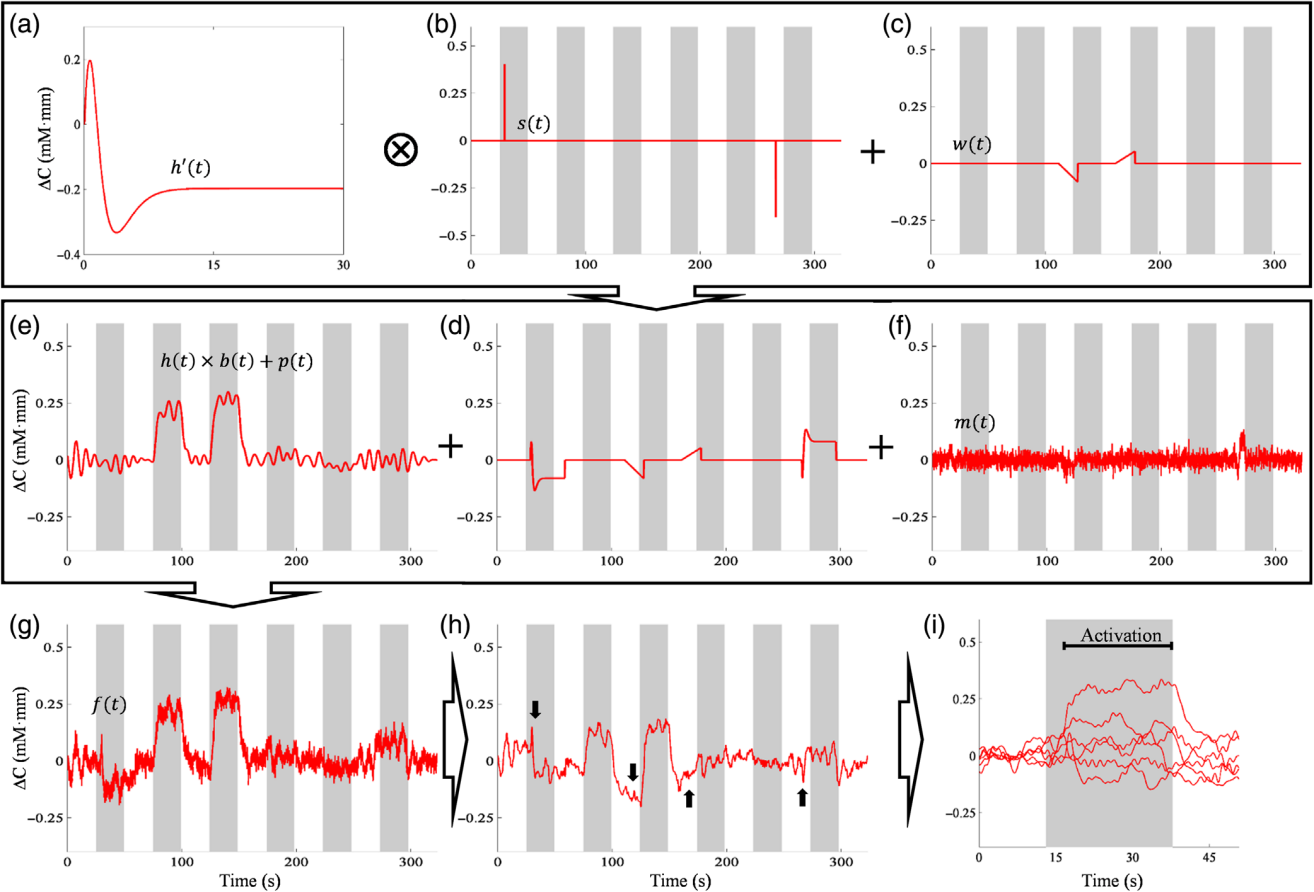
Generation of artificial model with synthetic noise models—spikes created by convolving the (a) first derivative HRF (b) with rebound step function and (c) epoch baseline shifting. (d) All noise models are composed together with task-related signal, (e) physiological, and (f) machine noise, and (g) resulting in fNIRS synthetic signal. After (h) preprocessing, (i) the epoch interval from continuous signal is extracted. Gray areas and black arrows represent the stimulus interval and modeled noises, respectively.

2. In the synthetic noise models (Fig. 6), noises were added as spikes and epoch baseline shifting. Spikes were generated by convolving the rebound step function [s(t), Fig. 6(b)] with the first derivative of the gamma function. To represent a nonrecovery spike, the first derivative gamma function [$h^{\prime }(t)$, Fig. 6(a)] was modified as follows:
  $$h^{\prime }(t)=\{h^{\prime }{\left(t\right)}_{t=1\to \max_{t}\;h^{\prime }(t)},[2\times h^{\prime }{\left(t\right)}_{t=\max_{t}\;h^{\prime }(t)\to \text{end}}]-\max_{t}\;h^{\prime }(t)\}.$$ (3)

The peak spike was varied within 0.14 to 0.48, and the spike direction could be either positive or negative. Epoch baseline shifting was simulated by adding either a positive or negative slope (μ=0.025  mM mm/s, σ=0 to 0.025  mM mm/s) in the baseline interval [w(t), Fig. 6(c)]. The number of noises (six at max. as epoch number), noise-type occurrences, and temporal locations were randomized. Those noises [Fig. 6(d)] were then computed together with convolved HRF-boxcar functions [h(t)×b(t)], physiological [p(t)], and machine [m(t)] noises. This resulted in the artificial task-response signal [f(t), Fig. 6(g)].

Both artificial signals (i.e., with and without synthetic noise models; N=1000 for each SNR variation) were preprocessed [see. 2.2, Figs. 5(g) and 6(h)] and the epochs were extracted. Epoch baselines (i.e., first 10 s) were normalized to zero amplitude [Figs. 5(h) and 6(i)]. To understand the relationship between the false negative rate (β) and rejection number, random epoch rejection (e.g., 0 to 4 trial rejection) was performed afterward. Furthermore, the significance (t-test) of epoch activation (i.e., average amplitude of signal 4 s after stimulus onset to end of stimulus) was evaluated in each artificial signal. Because the hemodynamic response change was set to be >0 on average, the detected insignificance presented the probability of a false negative rate.

### 2.6 Algorithm Feasibility

Algorithm feasibility was assessed in both artificial signals with synthetic noise models and real ADHD datasets. A dataset (N=98) containing artificial signals ($\frac{\mu }{\sigma }=1$) was constructed on the basis of the SNR distribution in the real dataset. The rejection algorithm was then applied to the artificial dataset using all noise criteria with associated optimization threshold ranges and acceptance rate by three epochs. This acceptance rate was set because three epochs were the lower boundary of remaining epochs in the real datasets. All criteria were used in this simulation because the artificial signals were created in accordance with these noise criteria. The criteria thresholds were optimized to obtain the lowest false negative rate from noisy artificial signals. Furthermore, the false negative rate and HRF recovery were compared between the results of optimum algorithm parameters and typical process without any rejection.

**Fig. 7:**
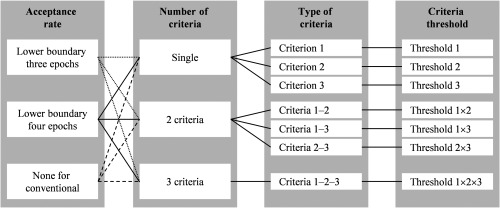
Optimization parameters including acceptance rate, number and type of criteria, and criteria threshold

Being different from the artificial dataset with controlled synthetic noise models, the prominent noise characteristics were unknown in the real datasets. Therefore, the algorithm application in the real datasets was optimized using four parameters: (1) acceptance rate (e.g., three and four epochs as lower boundaries), (2) number of criteria (e.g., single, two, and three criteria), (3) type of criteria (e.g., criteria 1, 2, and 3), and (4) criteria threshold (Fig. 7). Three and four epochs were selected as the range of acceptance rate because three epochs were the minimum number of remaining epochs in the real datasets, and >35% of ADHD data remained four epochs after visual rejection. In order to compare the concept of the current algorithm, conventional method without rejection limit was also performed with a varied number of criteria, type of criteria, and criteria threshold. In addition, we considered the possibility of task-dependent noise level. One task may be more prone to noise than another task. Therefore, the acceptance rate could be set differently in accordance with the task. All optimization parameters are summarized in Table 1.

**Table 1:** Combination list of optimization parameters.

Combination	Oddball task						GNG task					
	Acceptance rate			Number and type of criteria			Acceptance rate			Number and type of criteria		
	Three epochs	Four epochs	None	Criterion 1	Criterion 2	Criterion 3	Three epochs	Four epochs	None	Criterion 1	Criterion 2	Criterion 3
1		✓		✓	✓	✓		✓		✓	✓	✓
2		✓		✓	✓			✓		✓	✓	
3		✓		✓		✓		✓		✓		✓
4		✓		✓				✓		✓		
5		✓			✓	✓		✓			✓	✓
6		✓			✓			✓			✓	
7		✓				✓		✓				✓
8	✓			✓		✓	✓			✓		✓
9	✓			✓	✓		✓			✓	✓	
10	✓			✓	✓	✓	✓			✓	✓	✓
11	✓			✓			✓			✓		
12	✓					✓	✓					✓
13	✓				✓		✓				✓	
14	✓				✓	✓	✓				✓	✓
15			✓	✓		✓			✓	✓		✓
16			✓	✓					✓	✓		
17			✓	✓	✓	✓			✓	✓	✓	✓
18			✓	✓	✓				✓	✓	✓	
19			✓			✓			✓			✓
20			✓						✓			
21			✓		✓	✓			✓		✓	✓
22	✓			✓	✓	✓	✓			✓	✓	✓
23	✓			✓			✓			✓		
24	✓			✓		✓	✓			✓		✓
25	✓			✓	✓		✓			✓	✓	
26	✓				✓	✓	✓				✓	✓
27	✓						✓					
28	✓					✓	✓					✓
29		✓		✓	✓	✓		✓		✓	✓	✓
30		✓		✓	✓			✓		✓	✓	
31		✓		✓		✓		✓		✓		✓
32		✓		✓				✓		✓		
33		✓			✓	✓		✓			✓	✓
34		✓			✓			✓			✓	
35		✓				✓		✓				✓

While the algorithm optimization in the artificial dataset was oriented to increase HRF recovery, the algorithm optimization in the real datasets aimed to reproduce the former statistical results (Table 2) using the same datasets. According to previous studies,^23^^,^^24^^,^^40^^,^^41^^,^^52^^–^^55^ the attention and inhibition stimulus commonly brought significant activation defined as the increase of $\Delta C_{O2\mathrm{Hb}}$ (i.e., averaged amplitude of 4 s, end of stimulus) in the right inferior frontal gyrus/middle frontal gyrus (IFG/MFG), especially for TD children. Unlike cases for TD children, the IFG/MFG activation in ADHD children without medication was null. Administration of both MPH and ATX effectively modulated the IFG/MFG activation. Placebo was also administered; however, the IFG/MFG activation remained unchanged and greater effects were found in medicated conditions. Statistical inference was clarified as the observed significance (α=0.025; one-tail sample t-test). For example, optimization would be done well if no significance (p≥0.025) was found in preadministration (i.e., average of premedication and preplacebo) and postplacebo ADHD data and positive activation (p<0.025) was significantly observed in postmedication and intermedication ADHD data (see Table 2; column of training dataset I). Intermedication was described as medicated minus placebo-administered effects (i.e., postminus premedication/placebo).

**Table 2:** Significances of right IFG/MFG activation as training and validation targets.

Feasibility steps	1 Training	2 ADHD validation	3 TD validation	
Samples	22 ADHD (dataset I)	16 ADHD (dataset II)	22 ADHD (dataset I)	16 ADHD (dataset II)
Preadministration	p≥0.025	p≥0.025	p<0.025	p<0.025
Postmedication	p<0.025	p<0.025	—	—
Postplacebo	p≥0.025	p≥0.025	—	—
Intermedication	p<0.025	p<0.025	—	—

**Fig. 8:**
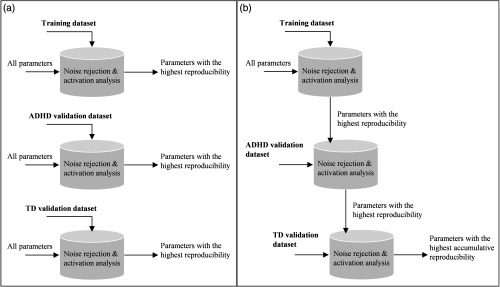
(a) Independent and (b) sequential optimizations for evaluating the robustness of algorithm’s parameters.

The algorithm was examined in three steps: (1) training in ADHD children samples (dataset I), (2) validation in ADHD children samples with different medications and performed tasks (dataset II), and (3) validation in TD children (datasets I and II) as a contrast sample. Successful validation likely indicated the feasibility of the algorithm across populations, tasks, and kinds of medication. Initially, each step was processed independently during optimization. However, the robustness of the algorithm parameters was also necessary to evaluate the capability of the algorithm for handling all datasets. Thus, the robustness analysis was performed sequentially by optimizing parameters that satisfied 100% reproducibility or produced the highest reproducibility in the training dataset. The algorithm parameters were called robust only if complete (i.e., 100%) reproducibility was obtained by the same parameters for all datasets. Figure 8 shows the manner in which independent and sequential optimizations were performed. In addition to the reproducibility of statistical inferences, the rejection accuracy (true positive and true negative between visual and current algorithm rejections) was investigated.

Because $\Delta C_{O2\mathrm{Hb}}$ is more pronounced compared to $\Delta C_{\mathrm{HHb}}$ signals ^56^^–^^58^ and the former results focused only on $\Delta C_{O2\mathrm{Hb}}$ results,^23^^,^^24^ the algorithm optimization was performed on $\Delta C_{O2\mathrm{Hb}}$ signals at IFG/MFG as a region-of-interest. To confirm the applicability of the algorithm in other signals, the rejection accuracy was also evaluated in $\Delta C_{\mathrm{HHb}}$ and $\Delta C_{\mathrm{Hb}\text{-}\text{total}}$ signals using the optimum parameters. However, the visual rejection process concerned only on $O_{2}\mathrm{Hb}$ signals. Therefore, the comparison between visual and adaptive rejection algorithms (see. Sec. 2.7) was performed following the rejection of $\Delta C_{O2\mathrm{Hb}}$ epochs for all signal types including $\Delta C_{\mathrm{HHb}}$ and $\Delta C_{\mathrm{Hb}\text{-}\text{total}}$.

### 2.7 Rejection Performances Through Visual Observation and Current Adaptive Algorithm

In order to evaluate the potential of the adaptive rejection algorithm to substitute for the visual rejection method, the rejection performances were compared to each another. The following three factors were examined. (1) We compared the temporal correlation (i.e., Pearson correlation) of resulting waveforms after visual and adaptive algorithm rejections. We also conducted an analysis of variance (ANOVA) in these temporal correlation coefficients to assess the feature of signal types ($\Delta C_{O2\mathrm{Hb}}$, $\Delta C_{\mathrm{HHb}}$, and $\Delta C_{\mathrm{Hb}\text{-}\text{total}}$) in algorithm performances. (2) The Spearman’s rank correlation of activation value after visual and adaptive algorithm rejections was also examined. The high correlation obtained indicated that both visual and adaptive algorithm rejections resulted in a similar tendency in activation value. However, the offset of activation value resulting from visual and adaptive algorithm rejections should be taken into consideration. Thus (3) statistical testing (one-sample t-test) was performed to assess whether a significant difference was found in activation values computerized by the two rejection methods. These comparisons were carried out for each sample type (i.e., TD, ADHD, preadministration, ADHD postmedication, and ADHD postplacebo) and each dataset (GNG or oddball tasks). Furthermore, these comparisons might be presented for each signal type if the result of the ANOVA was found significant.

## 3 Results

### 3.1 False Negative Rate and Algorithm Application in the Artificial Dataset

**Fig. 9:**
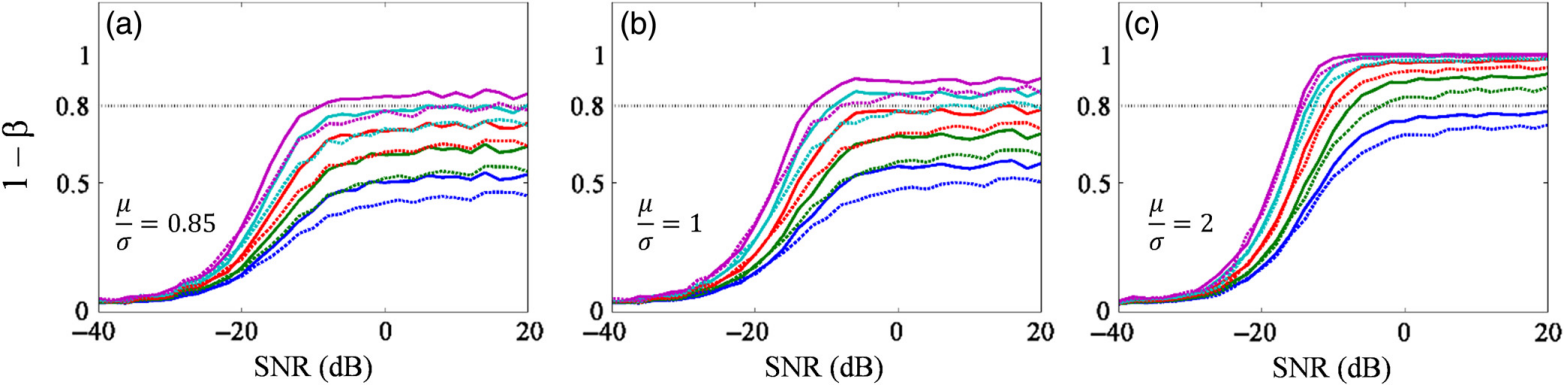
Rejection of random trials in simulated signals without (solid lines) and with (dotted lines) synthetic noise models in three ratios ($\frac{\mu }{\sigma }$ of activation) values: (a) 0.85 as similar as the average of real datasets (i.e., TD children with positive activation), (b) 1, and (c) 2 as the highest value in the real datasets of TD children. Colors—blue, green, red, cyan, and magenta indicate different rejection numbers up to four, three, two, one, and none rejected trials, respectively.

Figure 9 shows the effect of SNR, synthetic noise models, activation variance, and random epoch rejection on the statistical power (π; 1−β). According to the results, there were five notable observations. First, a low SNR brought low statistical power and a high false negative rate. Second, the statistical power reached a plateau as SNR improved more than −10 to −5  dB. Third, the occurrence of synthetic noise models (dotted lines in Fig. 9) decreased the statistical power particularly in a moderate-to-high SNR signal. Fourth, a high ratio between activation mean and standard deviation increased the statistical power, and an adequate power (i.e., 80%) could be obtained by less epoch samples. For example, five epochs [solid cyan line Fig. 9(a); a epoch rejection at maximum] were required at least to meet 80% power as $\frac{\mu }{\sigma }$ was 0.85. However, when $\frac{\mu }{\sigma }$ was set to 2, three epochs [solid green line Fig. 9(c); three epochs rejection at maximum] at minimum were sufficient to produce 80% power. Fifth, excessive rejection randomly performed did reduce the statistical power. In low SNR signals, lack of all effects on the statistical power was likely caused by dominating machine noises. Through this simulation, we confirmed that small epoch samples could not inflate a false negative rate if the rejection was done accurately and sufficiently, the signal quality was considerably high, and the activation phenomenon was prominent with low standard deviation.

**Fig. 10:**
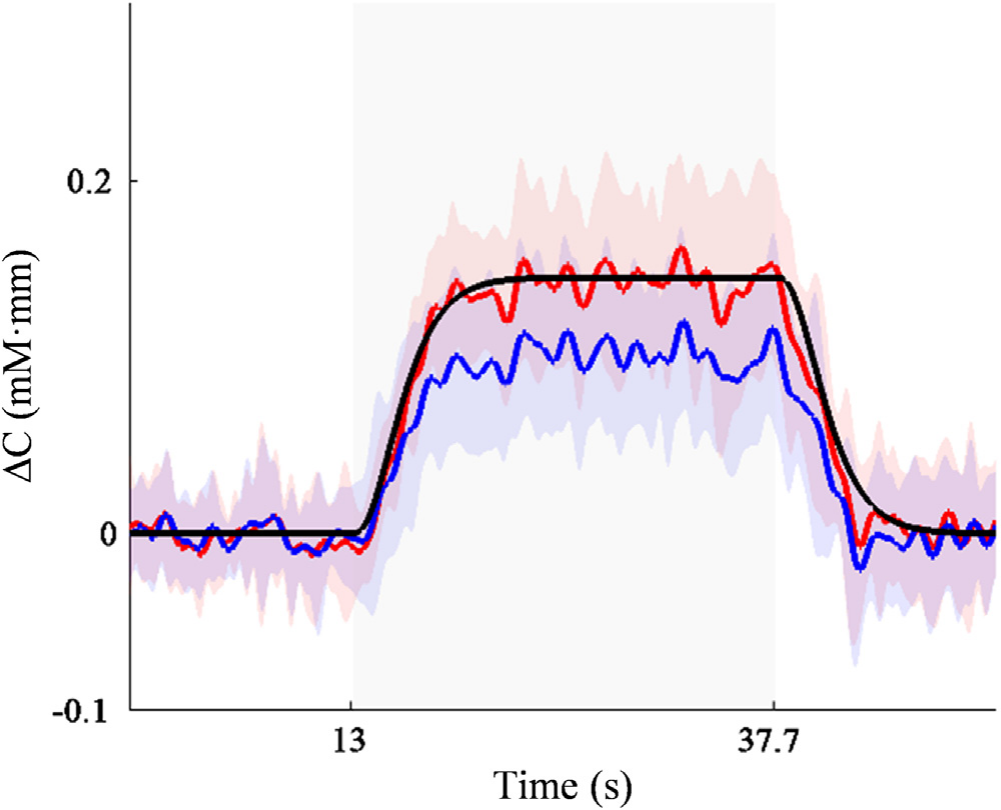
Recovery of HRF function (black plot) in the noisy simulated signals using the rejection algorithm (red plot). Blue plot indicated the average of noisy simulated signal without any rejection. Shaded patches represent the standard deviation.

In the next simulation, we optimized the algorithm by applying noisy artificial signals. We found the false negative rate (β) after algorithm rejection to be lower than that without any rejection (18%<34%). Figure 10 shows the HRF recovery result. The rejection of noisy epochs using the algorithm presented a better HRF recovery where the averaged signal (i.e., red plot in Fig. 10) was closely aligned with the HRF function (i.e., black plot in Fig. 10). Without any rejection, the HRF model could not be similarly retrieved particularly in the decreased activation amplitude. This was likely caused by the frequent occurrence (77%) of spikes severely affecting the baseline.

### 3.2 Optimization of Algorithm Parameters

**Fig. 11:**
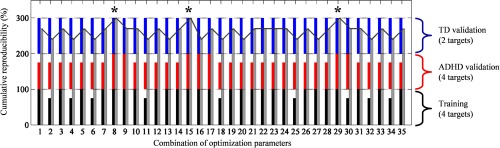
Optimization result for each algorithm combination (x-axis) aiming the complete reproducibility (y-axis) in all training (black bar), ADHD validation, (red bar), and TD validation (blue bar) as performing both independent and sequential optimizations (gray bar).

Figure 11 shows the results obtained for independent and sequential optimization. The x-axis indicates the combination of various algorithm parameters (e.g., acceptance rate, number and type of criteria, Table 1), whereas the y-axis illustrates the cumulative reproducibility level (i.e., summation of reproducibility level of training, ADHD validation, and TD validation datasets, 300% in total). Independent optimization with the maximum acquired reproducibility level was defined as black, red, and blue bar-plot for training, ADHD validation, and TD validation datasets, respectively. The cumulative reproducibility results of sequential optimization are shown in the gray bar-plot. According to Fig. 11, 26 combinations were able to completely reproduce statistical inferences (i.e., four targets, Table 2) of training dataset (black bar-plot). However, we found that the reduced reproducibility in the ADHD validation dataset (red bar-plot) was satisfied by seven combinations. The optimization performance was then improved in the TD validation dataset (blue bar-plot), in which all combinations achieved complete reproducibility. This result implied that the noise level and type might be different across datasets.

Despite this, we still found that independent optimization could result in complete reproducibility (i.e., 300%) for all datasets such as in combinations 8, 9, 15, 16, 17, and 29. However, only combinations 8, 15, and 29 (asterisks in Fig. 11) could apply the same criteria threshold for all datasets because of complete reproducibility in sequential optimization. On the other hand, the optimum criteria thresholds of combinations 9, 16, and 17 were limited in particular datasets. Therefore, combinations 8, 15, and 29 were more robust compared to combinations 9, 16, and 17. Even though both combinations 8 and 29 used all criteria (see Table 1), acceptance rate was uniformly set for all datasets in combination 8 as three epochs, whereas combination 29 employed different acceptance rates for datasets I and II (i.e., four and three epochs, respectively).

In order to compare the performance among combinations 8, 15, and 29, the rejection accuracy was evaluated using robust criteria’s thresholds. The rejection accuracy of datasets I and II was 67.2% and 64.2%, respectively, using combination 8. Combination 15 performed slightly worse than combination 8 with accuracies of 66.3% and 62.3% for datasets I and II, respectively. The rejection accuracy of combination 29 was superior to that of combinations 8 and 15; 74.5% and 69.2% in datasets I and II, respectively. Depending on the rejection accuracy, combination 29 was a more optimum (i.e., complete reproducibility and higher rejection accuracy) algorithm than combinations 8 and 15. Therefore, the results shown hereafter were analyzed using combination 29. The complete reproducibility of sequential optimization using combination 29 was acquired by setting criteria thresholds as 0.047 mM mm of amplitude change (criterion 1), 0.029  mM mm/s of baseline slope (criterion 2), and 0.6×IQR of correlation outlier (criterion 3). Using the optimum thresholds, we examined the rejection accuracy in $\Delta C_{\mathrm{HHb}}$ and $\Delta C_{\mathrm{Hb}\text{-}\text{total}}$ signals. In dataset I, we found rejection accuracy similar to that of $\Delta C_{O2\mathrm{Hb}}$ with 76% and 77% for $\Delta C_{\mathrm{HHb}}$ and $\Delta C_{\mathrm{Hb}\text{-}\text{total}}$ signals, respectively. The performance similarity was also confirmed in dataset II, in which the use of $\Delta C_{\mathrm{HHb}}$ $\Delta C_{\mathrm{Hb}\text{-}\text{total}}$ signals presented rejection accuracies of 75.6% and 69%. These results suggested the applicability of algorithm for any signal types.

### 3.3 Rejection Comparison Between Visual and Adaptive Algorithm

**Fig. 12:**
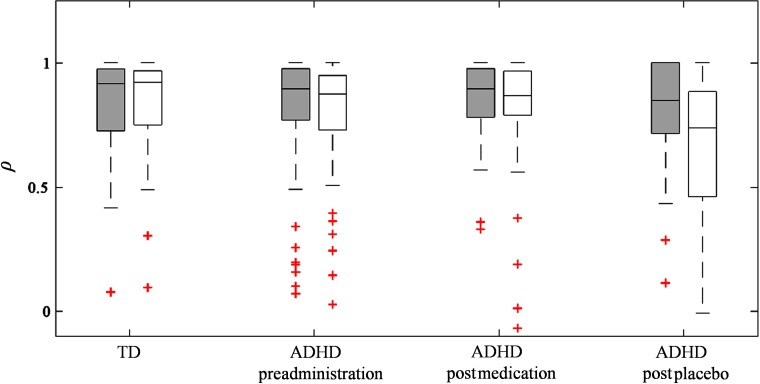
Box-plot of temporal correlation (ρ) between waveforms resulted by visual and adaptive algorithm rejections in datasets I (OB; filled boxes) and II (GNG; void boxes). Plus mark indicates data having substantial differences toward median value.

**Fig. 13:**
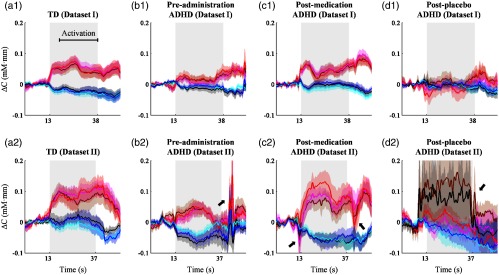
Subject-averaged waveforms without any rejection ($\Delta C_{O2\mathrm{Hb}}$, brown and $\Delta C_{\mathrm{HHb}}$, black plots), with visual rejection ($\Delta C_{O2\mathrm{Hb}}$, magenta and $\Delta C_{\mathrm{HHb}}$, cyan plots), and with adaptive rejection ($\Delta C_{O2\mathrm{Hb}}$, red and $\Delta C_{\mathrm{HHb}}$, blue plots) for (a1–a2) TD, (b1–b2) preadministration, (c1–c2) postmedication, and (d1–d2) postplacebo ADHD samples in (a1–d1) datasets I and (a1–d1) II. Shaded patches around plots are standard error while gray areas represent the stimulus interval.

Even though the adaptive rejection algorithm was able to completely reproduce all statistical inferences in all datasets with rejection accuracy about 70%, a comparison between visual observation and the adaptive rejection algorithm was comprehensively required. Waveforms ($\Delta C_{O2\mathrm{Hb}}$, $\Delta C_{\mathrm{HHb}}$, and $\Delta C_{\mathrm{Hb}\text{-}\text{total}}$) resulting from visual and adaptive algorithm rejections were temporally correlated. In prior experiments, we evaluated whether the signal types ($\Delta C_{O2\mathrm{Hb}}$, $\Delta C_{\mathrm{HHb}}$, and $\Delta C_{\mathrm{Hb}\text{-}\text{total}}$) brought significantly different correlation coefficients or not using Fisher z-transformation (i.e., neglecting ρ=1). ANOVA results showed no influence of signal type toward correlation in datasets I (F=0.74, p>0.05, and DF=2) and II (F=0.02, p>0.05, and DF=2). Therefore, the comparison indices were presented separately for each sample type (i.e., TD, ADHD preadministration, ADHD postmedication, and ADHD postplacebo) and each dataset (GNG or oddball tasks) regardless signal types. Figure 12 shows the boxplots of temporal correlations between the visual and algorithm results. Even though there were several outliers (i.e., the plus mark in Fig. 12), the median of correlation coefficient (r) for all sample groups was greater than 0.70 ($r_{\text{median}}=0.88\pm 0.06$). The maximum value 1 was also found, indicating that both visual observation and the adaptive algorithm rejected the same epochs. Furthermore, subject-average waveforms were compared in the raw data without any rejection and processed data using visual observation and the adaptive rejection algorithm as shown in Fig. 13. No obvious noise appeared in averaged waveforms for all sample groups in dataset I [Figs. 13(a1)–13(d1)]; however, preadministration and postplacebo ADHD samples of dataset II showed noisy averaged $\Delta C_{O2\mathrm{Hb}}$ and $\Delta C_{\mathrm{HHb}}$ waveforms in the raw data [arrows pointing to brown and black plots in Figs. 13(b2) and 13(d2)]. After performing visual rejection in dataset II, noise in postplacebo ADHD was successfully suppressed, yet a high amplitude change was still found in preadministration after stimulus interval [arrow pointing to magenta and cyan plots in Fig. 13(b2)]. The noise of preadministration ADHD samples in the dataset II [arrow pointing to red and blue plots in Fig. 13(b2)] was also observed after performing the adaptive rejection algorithm. Although the great dip [arrow pointing to magenta plot in Fig. 13(c2)] was observed in the baseline of postmedication ADHD samples in dataset II after visual rejection, the adaptive algorithm controlled by criterion 2 (shifted baseline) removed those noises. However, we observed a highly negative amplitude change in postmedication ADHD samples after the stimulus interval [arrow pointing to red plot in Fig. 13(c2)]. This amplitude decrease was seemingly seen in the raw data and visual rejection process with less amplitude change.

**Fig. 14:**
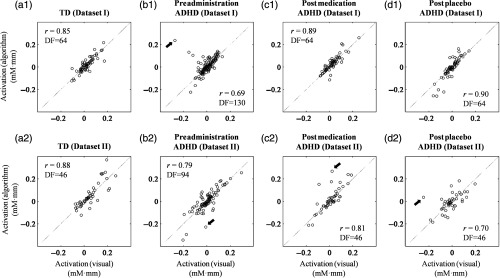
Scatter plot of activation values ($\Delta C_{O2\mathrm{Hb}}$, $\Delta C_{\mathrm{HHb}}$, and $\Delta C_{\mathrm{Hb}\text{-}\text{total}}$) computerized using visual (x-axis) and algorithm (y-axis) rejections for (a1–a2) TD, (b1–b2) preadministration, (c1–c2) postmedication, and (d1–d2) postplacebo ADHD samples in datasets (a1–d1) I and (a2–d2) II. Arrows indicate the examples of substantial offset between visual and adaptive algorithm activation values. r- and DF-values indicate the Spearman’s rank correlation coefficients (r) and the degrees of freedom (N−2).

Figure 14 shows the relationship between activation values resulting from visual and adaptive algorithm rejections. All correlation coefficients were significant (Spearman’s rank correlation, p<0.01). This confirmed that adaptive algorithm rejection brought about a similar tendency as that for visual rejection. However, several data were visually assessed as having great offsets from the diagonal line between visual and adaptive algorithm rejections, as shown in the arrows of Fig. 14. Furthermore, we statistically evaluated the offset from the diagonal line. The results showed that there were no significant differences (paired sample t-test, p≥0.05, Cohen’s d=0.03 to 0.23) between those offset values against zero in all datasets.

## 4 Discussion

This study aimed to achieve an approach in managing noisy fNIRS data by adaptively identifying and rejecting noises in individual data. We also introduced the concepts of acceptance rate (i.e., minimum rejection rate) and quantitative data ranking depending on noise level to determine rejections and maintain the statistical sample number. By applying those concepts, the noise identification was not limited by the preset noise criteria and yet became more versatile in interpreting noises on an individual data scale. To evaluate the feasibility of our idea, we performed a simulation of synthetic noisy signals and used datasets previously analyzed (i.e., ADHD-TD datasets) to tune the rejection algorithm while aiming to reproduce former statistical inferences. We prepared three noise criteria (sudden amplitude change with recovery failure, extremely high/low baseline’s slope, and low interepoch correlation) and determined the acceptance rate (i.e., ≥three, four epochs). Afterward, the rejection algorithm selected the noise criteria to be adopted and parameters based on data-driven optimization. Our algorithm of personal rejection control was proven effective through the simulation result. The algorithm demonstrated the advantages in HRF recovery and achieving a low false negative rate in the artificial dataset with varied noise severity (i.e., SNR). The algorithm application in the real datasets successfully found robust sets of rejection criteria for all sample groups (i.e., different population, condition, and interference). They were sudden amplitude change with recovery failure by 0.047 mM mm, extremely high/low baseline’s slope above 0.029  mM mm/s, and low interepoch correlation summation value below 0.6×IQR. Even though three noise criteria were made available, the usage of noise criteria in determining rejection depended on individual noise level and data characteristic. One or more criteria might be excessively strict in performing data rejection; therefore, the remaining criteria or even the consideration of data noise ranking handled the rejection. The adaptive rejection algorithm achieved the complete reproducibility of former statistical inferences and similar performance compared to visual rejection. Thus the adaptive rejection algorithm can be a potential substitute for the visual rejection method.

### 4.1 Noise Correction Versus Adaptive Rejection Algorithm

In addition to the noise rejection, noise correction has been persistently attempted in fNIRS studies. Previous studies have shown the comparison over several correction methods in analyzing various datasets.^14^^,^^15^^,^^19^^–^^21^ All studies reported the superiority of correction methods in managing noises compared to rejection methods with respect to maintaining sufficient sample number.

However, there are three arguments as to why we did not consider the noise correction method in the current datasets. First, the noise correction method also experiences temporal data loss in a way similar to that of the rejection method. The true activation confined in dominant noises might be corrected and the risk of true activation elimination together with noises will likely increase. Therefore, the data pattern may substantially change compared to the raw data as a result of over-processing. Jahani et al.^21^ reported the advantage of hybrid methods (e.g., a combination of two or more correction methods) in reducing artifacts. Instead of remarkable performances, the waveform significantly changed. Principal component analysis (PCA) has also been reported to result in unstable performances.^14^^,^^20^ The parameter of component selection (e.g., 80% variance elimination) may arbitrarily remove the recovered activation or retain noises. Furthermore, multichannel measurement is required with the assumption of uncorrelated components.^59^^–^^61^ In addition, this basic assumption will likely be violated when the true activation and noises are convolved to each other. Other than PCA, independent component analysis (ICA) has been widely used to remove nonneuronal components (i.e., motion artifact, extracerebral, and physiological interferences).^62^^–^^64^ Aarabi and Huppert^65^ presented the advantage of the ICA method in decomposing the physiological noises even from single channel. However, the preprocessing still required multichannel measurement to reduce motion artifact using spatial-ICA. The ICA performance highly depends on the multichannel measurement; reduced efficiency happens when the number of channels is less than those of independent sources.^66^ The number of sources are unknown, and the sources (true activation and noises) themselves are likely to correlate with each other. These become limitations of ICA. Even though attempts have been made to apply ICA to real-time applications,^67^ there are some constraints related to limited data, limited analysis time, and dynamic changes of brain signals.^68^ Second, another concern is the current dataset including data from disordered children, which we would like to understand comprehensively. Performances of noise correction had been compared based on the HRF recovery parameter.^14^^,^^69^ This parameter may be invalid for these datasets and the risk of over-processing may conceal the true characteristic of disordered samples. Third, the noise feature cannot be interpreted easily and each correction method may be suitable for particular noise characteristics. Thus the correction performance results varied depending on datasets. Spline interpolation is useful for eliminating apparent noise such as high-frequency spikes; however, it has a limitation in detecting overlapped noises with true activation signals.^18^^–^^20^ The performance is highly related to individual noise level and the method’s parameters should be adjusted individually for optimum performance.^19^^,^^70^

As discussed above, the correction methods have three disadvantages: temporal data loss leading to substantial waveform change, subjective activation assumption, and noise-dependent performance. Such problems sometimes pose difficulties in assessing fNIRS data, especially those for disordered children. The current adaptive rejection algorithm offers controlled data loss with maintained data originality and individual-personalized assessment. Therefore, it addresses the disadvantages of noise correction and provides a practical alternative for treating noise-prone children data.

### 4.2 Optimum Algorithm’s Parameters

After the rejection algorithm was optimized, all criteria were found to be optimum as we had expected. Among the three noise characteristics, criterion 1 is the most obvious noise characteristic that could be identified with bare visual observation. However, the complete reproducibility results achieved only using all criteria with an adaptive concept may confirm that visual judgment is prone to subjectivity each time individual data are assessed while maintaining the sample number. The necessity of all criteria in the rejection algorithm confirmed that the noise characteristics varied from extreme signal amplitude change to slow change of baseline. The occurrence of each noise characteristic varied across individuals. Even though the algorithm parameters were optimized using $\Delta C_{O2\mathrm{Hb}}$ signals, the algorithm applicability in $\Delta C_{\mathrm{HHb}}$ and $\Delta C_{\mathrm{Hb}\text{-}\text{total}}$ was confirmed with comparable rejection accuracies. Furthermore, we examined the effect of preprocessing that could affect the algorithm parameters. However, through our confirmation of the real dataset characteristics, only criterion 1 was significantly influenced by preprocessing step. Therefore, the preprocessing caused the change in the criterion 1 threshold, which should be tuned. Including all criteria enables this adaptive rejection algorithm to be useful for any signal types in both datasets with differing noise types and levels.

Our first hypothesis suggested that there was the possibility of different noise levels depending on performed tasks. Therefore, both independent and sequential optimizations were performed depending on the task. Even for the same noise criteria, we found that complete reproducibility was obtained from different acceptance rates: the GNG dataset required an acceptance rate (i.e., ≥three epochs) lower than that of the OB dataset (i.e., ≥four epochs). However, this difference may not be attributed to types of performed tasks. We observed the complete reproducibility of TD samples in the GNG dataset by setting four epochs of acceptance rate as in the OB dataset. On the other hand, ADHD samples in the GNG dataset (i.e., the red bar-plot in Fig. 11) only reached complete reproducibility when the acceptance rate was set to be either three epochs or none (i.e., nonadaptive rejection). According to Figs. 13(b2)–13(d2), ADHD preadministration and postplacebo samples of the GNG dataset were substantially noisy compared to TD samples of the GNG dataset [Fig. 13(a2)]. Therefore, our hypothesis of the task-dependent noise level could not be supported, and instead, the acceptance rate was rather influenced by the individual noise level of a particular subject group being analyzed.

### 4.3 Visual Rejection Versus Adaptive Rejection Algorithm

We assessed three factors in comparing visual and adaptive algorithm rejections and all of them provided positive support for the algorithm potential as a substitute for visual rejection. We observed similar waveforms and activation tendencies (i.e., factors 1 and 2) between visual and adaptive algorithm rejection. Furthermore, we found no significant differences in activation results for either method. Compared to the nonadaptive algorithm with rejection up to >90%,^14^ the current adaptive algorithm is able to preserve the epoch number as an acceptance rate. In addition to the capability of maintaining data volume, it provides other advantages such as analysis speed, less subjectivity, and higher applicability for even inexperienced data analysts.

We should note here that we observed several discrepancies between visual and algorithm-based rejections such as the substantial offsets indicated by arrows in Fig. 14. There are two arguments related to the reasons for these offsets. First, noises with very slow oscillation periodically become out of phase from the stimulus. This characteristic violated criteria 2 and 3 of shifted epoch’s baseline and low interepoch correlation. Although the algorithm likely identified noisy epochs more than the acceptance rate, they might have remained nonrejected to maintain the minimum sample number. Second, severe noises such as intermittent sudden amplitude increase and decrease and thus affect the whole measurement interval. This data likely had lower signal quality compared to other data. The above arguments indicate that the adaptive algorithm has limitations in mimicking the visual rejection performance, particularly in data with high interepoch variability and bad signal quality. In addition, it should be noted that perfect reproducibility of visual rejection results may not always be desired as visual inspection necessarily entails subjective evaluation. Given the moderately high reproducibility in this study, further validation of the adaptive rejection algorithm should be performed from different perspectives.

### 4.4 Limitations

In spite of the favorable results obtained with the adaptive rejection algorithm, we still found three limitations. The first was the rejection accuracy toward visual rejection, which was on average about 73.5% (i.e., the robust criteria’s threshold) in both datasets for all signal types. We still consider that this is a fairly good achievement compared to the accuracy that skillful analysts produce in performing visual rejections. Further countermeasures for collecting more data will be necessary for finer algorithm tuning. The second limitation relates to the great difference in activation values produced by visual and adaptive algorithm rejections for several types of data (i.e., substantial offsets). The adaptive algorithm does not perform very well in evaluating data with extremely bad quality, such as sudden amplitude changes repetitively occurring during measurements and high interepoch variability because of slow and out-of-phase oscillation toward stimulus. This showed us that bad-quality data should not be used. We, therefore, feel that applying the algorithm in the future to real-time measurements may mitigate the current limitation.

Even though out-of-phase signals toward the stimulus may indicate noises, we should be careful in interpreting this phenomenon. Previous studies have reported the high interepoch variability in ADHD datasets.^71^^–^^74^ Therefore, noise criterion 3 (i.e., low interepoch correlation) might not be appropriate for current datasets. In order to examine this possibility, reoptimization of noise criteria is required with wider data sets.

The third limitation is the necessity of parameter (i.e., criteria type, threshold, and acceptance rate) retuning if datasets have completely different signal qualities, measurement instruments (i.e., criteria thresholds), and imbalance paradigm intervals (i.e., criterion 3). The different noise occurrences and signal quality have been explained above with the examples of datasets I and II causing different optimum acceptance rates. The sensitivity of criteria thresholds (e.g., criteria 1 and 2) will likely decrease when the frequency sampling is less than the optimized datasets (and *vice versa*). Furthermore, criterion 3 was developed on the basis of the block-design study with constant paradigm intervals; however, it is unsuitable for experimental design with imbalance paradigm intervals and resting-state measurements. Criterion 1 (and 2) might be sufficient to control false negative rates in those datasets. If the algorithm performance apparently becomes worse, the typical noise in the particular datasets should be observed further to replace the current criteria. Despite these limitations, the bottom-up concept of the algorithm is still promising, enabling users to easily adjust the number of noise criteria.

## 5 Conclusion

We have proposed an approach to manage motion artifacts in fNIRS data sets using an adaptive algorithm for guaranteeing an acceptance rate (e.g., ≥three epochs). Three predetermined noise criteria (i.e., sudden amplitude change, shifted baseline, and minimum intertrial correlation) are adopted in this algorithm to adaptively reject and select the least noisy trials (epochs) maintaining the sufficiency of trial numbers, which is an issue in conventional rejection methods. Real fNIRS data obtained during a cognitive task was applied to the algorithm and allowed us to conclude the algorithm was feasible by showing the complete reproducibility obtained with it, referring to prior ADHD studies. The criteria’s parameters were also found to be robust and valid for both training and validation datasets.

Although this study presented the first optimized parameters for the noise criteria, there will always be room for improvement such as noise criteria optimization and iterative parameter tuning. The usage of the adaptive algorithm is not limited to postmeasurements. It can also be used to evaluate real-time noise after completing a number of repetitions that is at least similar to the acceptance rate. This will enable the data acquisition quality to be improved and the risk of algorithm mistuning due to low data quality to be reduced. We believe this adaptive algorithm represents a concept in fNIRS data preprocessing and that it will prove to be highly resourceful for many applications.
